# Publisher’s Note: A New Chapter for *Hematology Reports*—Continued Publication by MDPI

**DOI:** 10.3390/hematolrep14010001

**Published:** 2022-01-30

**Authors:** Liliane Auwerter

**Affiliations:** MDPI, St. Alban-Anlage 66, CH-4052 Basel, Switzerland; liliane.auwerter@mdpi.com

*Hematology Reports* published its first volume in 2009, which included 10 publications: 8 original articles and 2 reviews [1]. The journal accepts submissions of original articles, case reports, and reviews focused on molecular and cell biology, genetics, and the pathophysiology and epidemiology of blood disorders, as well as controlled trials on blood research.

In September 2019, *Hematology Reports* became the official journal of the Society of Hematologic Oncology Italy (SOHO Italy). The journal publishes their scientific material from conferences and other events. The presidents of SOHO Italy are Dr. Claudio Cerchione, Prof. Dr. Giovanni Martinelli, and Prof. Hagop Kantarjian (Director of Leukemia Department of MD Anderson Cancer Center, Houston, Texas, USA) [2]. 

As of 2022, *Hematology Reports* will be published by MDPI and will continue to serve the scientific community by publishing high-quality, open-access, peer-reviewed articles on all aspects of the prevention, diagnosis, and management of disorders of the blood. 

We would like to offer a warm welcome to all Editorial Board Members, SOHO society members, and authors. We hope *Hematology Reports* will continue to publish high-quality and interesting research in the field of hematology.
